# Supplementary material for: FERN – a Java framework for stochastic simulation and evaluation of reaction networks
Source: BMC Bioinformatics. 2008 Aug 29;9:356. doi: 10.1186/1471-2105-9-356 (PMC2553347; doi:10.1186/1471-2105-9-356)
Supplement: Additional file 1 — FERN distribution, Version 1.3. This archive contains the FERN source code and binaries as well as documentation and example models in FernML and SBML. [file 1471-2105-9-356-S1.zip › fern/doc/javadoc/fern/tools/ColorSpectrum.html]

ColorSpectrum


---


|  |  |  |
| --- | --- | --- |
| **Overview** \| **Package** \| **Class** \| **Use** \| **Tree** \| **Deprecated** \| **Index** \| **Help** | |  |
| PREV CLASS   **NEXT CLASS** | **FRAMES**    **NO FRAMES**     **All Classes** |  |
| SUMMARY: NESTED \| FIELD \| CONSTR \| METHOD | DETAIL: FIELD \| CONSTR \| METHOD |  |


---


## fern.tools Class ColorSpectrum

```
java.lang.Object
  fern.tools.ColorSpectrum
```

---

``` public class ColorSpectrum extends Object ```

Provides methods to assign colors to numbers between 0 and 1.

**Author:**
:   Florian

---

**Constructor Summary**

`ColorSpectrum()`


| **Method Summary** | |
| --- | --- |
| `static Color` | `getGrayScaleSpectrum(float value)`             Gets a gray scale color representing the given number, 0 means white, 1 means black. |
| `static Color` | `getSpectrum(float val, Color... colors)`             Gets a color representing the given number in an given color scale. |

**Methods inherited from class java.lang.Object**

`clone, equals, finalize, getClass, hashCode, notify, notifyAll, toString, wait, wait, wait`

**Constructor Detail**

### ColorSpectrum

```
public ColorSpectrum()
```


**Method Detail**

### getGrayScaleSpectrum

```
public static Color getGrayScaleSpectrum(float value)
```

:   Gets a gray scale color representing the given number, 0 means white, 1 means black.

    :   **Parameters:**: `value` - between 0 and 1 **Returns:**: gray scale color

---


### getSpectrum

```
public static Color getSpectrum(float val,
                                Color... colors)
```

:   Gets a color representing the given number in an given color scale.
    You can give as much colors as you want. The resulting color will be interpolated.

    :   **Parameters:**: `val` - between 0 and 1: `colors` - color spectrum **Returns:**: color for the value


---


|  |  |  |
| --- | --- | --- |
| **Overview** \| **Package** \| **Class** \| **Use** \| **Tree** \| **Deprecated** \| **Index** \| **Help** | |  |
| PREV CLASS   **NEXT CLASS** | **FRAMES**    **NO FRAMES**     **All Classes** |  |
| SUMMARY: NESTED \| FIELD \| CONSTR \| METHOD | DETAIL: FIELD \| CONSTR \| METHOD |  |


---
